# Hybrid female sterility due to cohesin protection errors in mouse oocytes

**DOI:** 10.1126/sciadv.adx9729

**Published:** 2026-02-04

**Authors:** Warif El Yakoubi, Bo Pan, Takashi Akera

**Affiliations:** Cell and Developmental Biology Center, National Heart, Lung, and Blood Institute, National Institutes of Health, Bethesda, MD 20894, USA.

## Abstract

Hybrid incompatibility can lead to lethality and sterility of F1 hybrids, promoting speciation. The cell biological basis underlying hybrid incompatibility remains largely unknown, especially in mammals. Here, we found that female hybrids between *Mus musculus domesticus* and *Mus spicilegus* mice are sterile due to the failure of homologous-chromosome separation in oocyte meiosis, producing aneuploid eggs. This nondisjunction phenotype was driven by the mislocalization of the cohesin protector, SGO2, along the chromosome arms instead of its typical centromeric enrichment, resulting in cohesin overprotection. The upstream kinase, BUB1, showed a higher activity in hybrid oocytes, explaining SGO2 mistargeting. Higher BUB1 activity was not observed in mitosis, consistent with viable hybrid mice. Cohesion defects were also evident in hybrid mice from another genus, *Peromyscus*, wherein cohesin protection is weakened. Defective cohesion in oocytes is a leading cause of reduced fertility. Our work provides evidence that a major cause of human infertility may play a positive role in mammalian speciation.

## Teaser

Misregulation of chromosome cohesion during female meiosis serves as a reproductive isolating barrier in mice.

## INTRODUCTION

Postzygotic reproductive isolation refers to the inability of one population to produce viable and fertile offspring with another population or species. A key objective in evolutionary biology is to uncover the mechanisms driving hybrid incompatibilities, which can act as reproductive isolating barriers (*1*–*7*). Prior studies have identified hybrid incompatibility in chromosome condensation in oocytes from hybrid mice between *Mus musculus domesticus* (hereafter *domesticus*) and *Mus spretus*, resulting in condensation defects and female subfertility (*8*–*10*). Chromosome condensation is governed by the condensin complex, a member of the SMC (structural maintenance of chromosomes) family of complexes that regulate chromosome structure and dynamics (*11*, *12*). SMC complexes are ancient enzymes that have undergone substantial evolution in metazoans (*13*), leading us to hypothesize that their misregulation (and more broadly chromosome misregulation) might represent a common species barrier.

## RESULTS

**Fig. 1.**
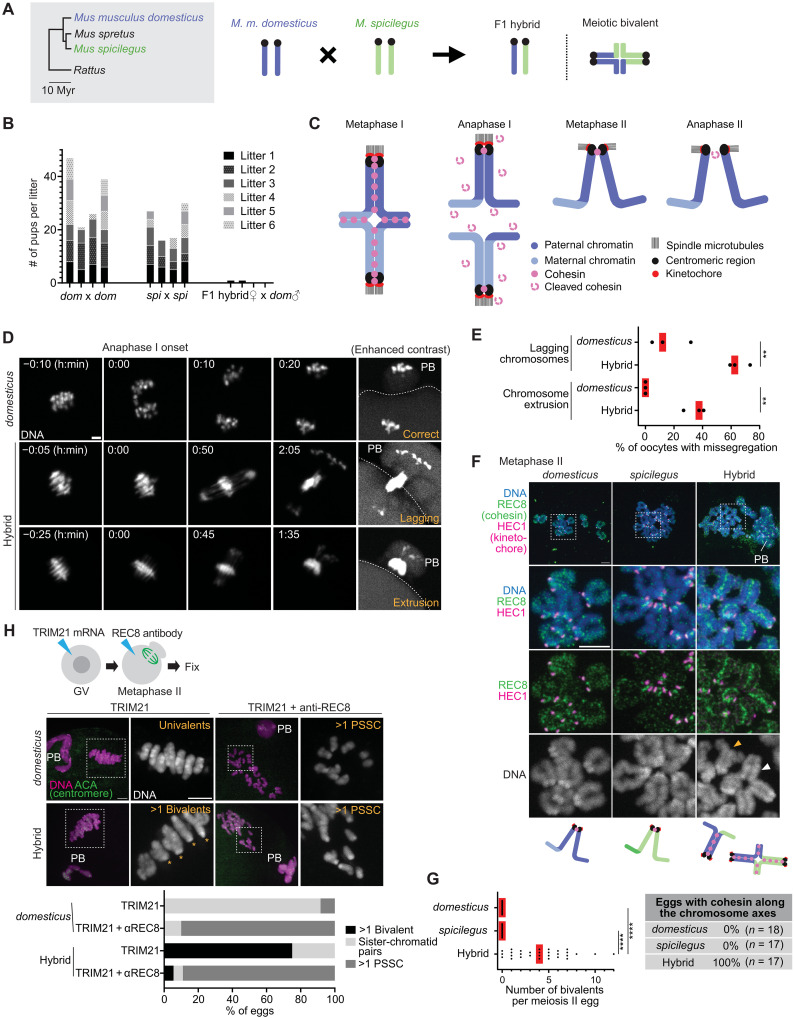
Cohesin misregulation drives nondisjunction in hybrid oocytes. (**A**) Phylogenetic tree of mouse species [Myr (million years)] and schematic of the hybrid mouse system. (**B**) Six-month fertility test: age-matched *domesticus* (*dom*), *spicilegus* (*spi*), and F1 hybrids were used. The number of pups per litter for each breeding cage was quantified (four cages per genotype). Two F1 females delivered one pup, which died shortly after birth. (**C**) Schematic of meiosis. (**D**) DNA was visualized in *domesticus* and hybrid oocytes with SPY650-DNA or by expressing separase biosensor, H2B-mScarlet-Rad21-mNeonGreen (Fig. 2A) to live-image anaphase I; mScarlet images are shown; PB, polar body; h, hours; dashed lines, oocyte cortex. (**E**) Proportion of oocytes with lagging chromosomes or chromosome extrusion in (D) was quantified (*n* = 68 and 38 oocytes for *domesticus* and hybrid, respectively); each dot in graph represents one experiment; red lines, mean; unpaired two-tailed *t* test was used for statistics; **P < 0.01. (**F**) Chromosome spreads using *domesticus*, *spicilegus*, and hybrid metaphase II eggs were stained for HEC1 and REC8; orange arrowhead, sister-chromatid pairs; white arrowhead, bivalents. (**G**) Number of bivalents per meiosis II egg (*n* = 22, 17, and 30 eggs for *domesticus*, *spicilegus*, and hybrid) and percentage of eggs with cohesin along chromosome axes (*n* = 18, 17, and 17 eggs for *domesticus*, *spicilegus*, and hybrid) were quantified using the images in (F); each dot in the graph represents one egg; red line, median; ****P < 0.0001. (**H**) *domesticus* and hybrid oocytes microinjected with mCherry-TRIM21 mRNA and REC8 antibody were fixed at metaphase II and stained for ACA; asterisks, bivalents. Percentage of meiosis II eggs with >1 bivalent, >1 PSSC, and sister-chromatid pairs was quantified (*n* = 12, 10, 16, and 18 eggs for *domesticus* TRIM21, *domesticus* TRIM21 + anti-REC8, hybrid TRIM21, and hybrid TRIM21 + anti-REC8). Scale bars, 5 μm. Schematics in (C) and (F) were created in BioRender. El Yakoubi, W. (2025) https://BioRender.com/wa90bys.

To explore this hypothesis, we examined hybrid females resulting from crosses between *domesticus* and *Mus spicilegus* (hereafter *spicilegus*), another closely related species capable of producing viable F1 hybrids in both directions (although *spicilegus* primarily served as the sire except for fig. S1C) (Fig. 1A) (*14*–*17*). We found that *domesticus* x *spicilegus* hybrid females are sterile (Fig. 1B), prompting us to investigate their oocytes for potential chromosome misregulation. Hybrid males are aggressive, and we have not been able to examine their fertility.

**Fig. 2.**
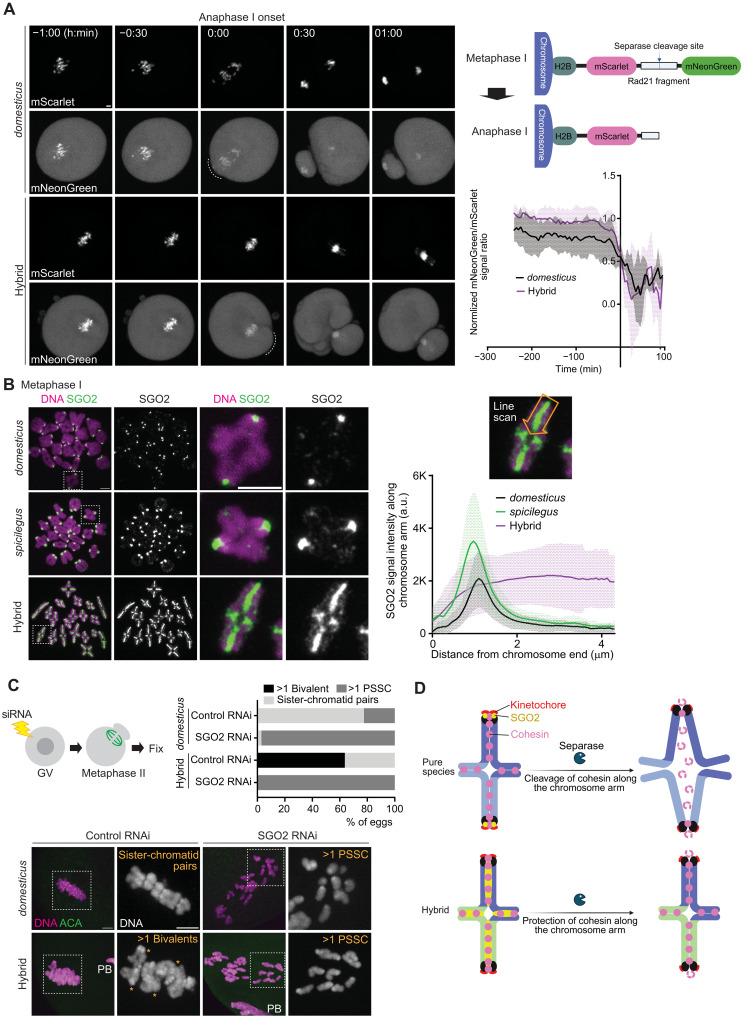
Cohesin overprotection by the ectopic targeting of SGO2. (**A**) Schematic of the separase sensor construct (top right). *domesticus* and hybrid oocytes expressing the separase sensor were imaged from metaphase I to anaphase I (left). The ratio of mNeonGreen signals divided by mScarlet signals on the chromosomes was quantified over the time course (bottom right, *n* = 7 and 7 oocytes for *domesticus* and hybrid, respectively); line graph shows the mean values of the mNeonGreen/mScarlet ratio with the shaded regions representing SD. (**B**) Chromosome spreads were performed at metaphase I using *domesticus*, *spicilegus*, and hybrid oocytes and stained for SGO2. Line scans of SGO2 signals were performed along the chromosome arm starting from the chromosome end with a centromere. Line graph shows the mean values of SGO2 intensities along the chromosome arm with the shaded regions representing SD (*n* = 94, 91, and 150 chromosomes for *domesticus*, *spicilegus*, and hybrid). a.u., arbitrary units. (**C**) *domesticus* and hybrid oocytes electroporated with control or SGO2 small interfering RNA (siRNA) were fixed at metaphase II and stained for ACA. Asterisks in the images denote bivalents. The percentage of eggs with >1 bivalent, >1 PSSC, and normal sister-chromatid pairs were quantified (*n* = 58, 68, 11, and 17 eggs for *domesticus* + control RNAi, *domesticus* + SGO2 RNAi, hybrid + control RNAi, and hybrid + SGO2 RNAi). Scale bars, 5 μm. (**D**) Model of hybrid incompatibility in cohesin protection, leading to hybrid female sterility. SGO2 localizes to the centromere and protects cohesin at the pericentromere in pure species oocytes. In contrast, SGO2 ectopically localizes along the arms in hybrid oocytes, protecting cohesin along the arms and causing nondisjunction of homologous chromosomes. Schematics in (A) and (D) were created in BioRender. El Yakoubi, W. (2025) https://BioRender.com/wa90bys.

To understand the mechanisms causing sterility in hybrid females, we imaged chromosome dynamics live from metaphase I to anaphase I in hybrid oocytes (Fig. 1, C and D). During meiotic prophase, homologous chromosomes pair and recombine, forming meiotic bivalents that segregate at anaphase I, becoming sister-chromatid pairs in meiosis II (Fig. 1C) (*18*–*21*). Anaphase I onset is indicated by cell membrane protrusions, eventually leading to the polar body formation (Fig. 1D, see dashed lines in Fig. 2A for the membrane protrusion at anaphase I onset). In pure *domesticus* oocytes, anaphase I onset coincided with the separation of bivalents (Fig. 1D and movie S1). However, in hybrid oocytes, bivalents failed to segregate properly at anaphase I onset (Fig. 1, D and E, and movies S2 and S3). Missegregated chromosomes were either trapped between the egg and the polar body (lagging chromosomes) or extruded to the polar body (chromosome extrusion). Chromosome missegregation was followed by cytokinetic failures, producing polar bodies of varying sizes (fig. S1A). These severe meiotic errors explain the sterility of hybrid females.

To determine the cause of this missegregation, we analyzed chromosome morphology in metaphase II eggs. In pure species, chromosomes were all sister-chromatid pairs, indicating successful separation of homologous chromosomes at anaphase I (Fig. 1, F and G). In contrast, hybrid meiosis II eggs contained multiple bivalents [Fig. 1, F (hybrid, white arrowhead) and G, and fig. S1B]. Meiosis II eggs with bivalents are aneuploid, as resulting embryos would have three copies of the chromosome, leading to embryonic lethality (*22*, *23*). These observations suggest that defective separation of bivalents in hybrid oocytes results in chromosome missegregation, egg aneuploidy, and infertility.

We hypothesized that the defective homolog separation might be due to misregulation of cohesin, an SMC complex responsible for holding sister chromatids together during mitosis and meiosis (*24*–*27*). Cohesin is removed from chromosomes in two steps during meiosis: It is initially loaded along chromosome arms during premeiotic S-phase and cleaved by separase at anaphase I, except for the pericentromeric pool, which is crucial for chromosome biorientation on the meiosis II spindle (Fig. 1C) (*28*–*31*). We analyzed the localization pattern of a meiosis-specific cohesin subunit, REC8, at metaphase II (Fig. 1F) (*24*). In pure species, REC8 cohesin was localized between sister kinetochores, connecting sister chromatids as previously reported (*32*–*34*). In hybrid oocytes, REC8 signals were present between sister chromatids along the entire arms of unseparated bivalents (Fig. 1, F and G). Sister-chromatid pairs in hybrid eggs also showed similar REC8 distribution along the arms, which is consistent with the observation that sister chromatids retained attachments along their lengths unlike typical ones connected exclusively at the pericentromere (Fig. 1F, hybrid, orange arrowhead). Similar REC8 localization and defective homolog separation were observed in hybrid females produced by the reciprocal cross (i.e., *spicilegus* female x *domesticus* male) (fig. S1C), suggesting that the phenotypes are not due to maternal or paternal effects. To confirm that defective homolog separation is due to remaining REC8 on chromosomes, we depleted REC8 using the Trim-Away method (Fig. 1H) (*35*, *36*). REC8 degradation resulted in chromatid separation in both *domesticus* and hybrid eggs, indicating that REC8 cohesin holds homologs together in hybrid eggs (rather than DNA catenation, etc.).

Aging reduces cohesin levels on chromosomes in mammalian oocytes (*37*–*39*). We wondered whether reduced cohesin levels in aged oocytes might facilitate homolog separation in hybrid oocytes. Consistent with this idea, meiosis II eggs from aged hybrid females had fewer bivalents than those from younger hybrid females (fig. S2). This observation supports the idea that the nondisjunction phenotype is caused by cohesin remaining on chromosomes.

Cohesin on chromosome arms is phosphorylated during meiosis I, permitting separase-mediated cleavage and homologous chromosome segregation at anaphase I (*40*–*42*). Cohesin at the centromere region is dephosphorylated by the Shugoshin (SGO)–Protein Phosphatase 2A (PP2A) (SGO-PP2A) complex, protecting it from separase and maintaining sister-chromatid cohesion until metaphase II (*43*–*45*). Oocytes with inactive separase fail to separate homologous chromosomes at anaphase I, proceeding to metaphase II with bivalents (*31*, *46*). Similarly, high SGO-PP2A activity leads to overprotection of cohesin along arms, causing defective homolog separation (*31*). On the basis of these studies, we propose two possible models to explain the defective homolog separation in *domesticus* x *spicilegus* hybrid oocytes: (i) The separase activity is lower in the hybrid, preventing homolog separation, or (ii) the SGO-PP2A activity is higher in the hybrid, resulting in cohesin overprotection and defective homolog separation.

To test the first possibility, we used a separase activity sensor (Fig. 2A), which is an ideal tool to measure the separase activity during mitosis and meiosis (*47*–*49*). This sensor construct is composed of a separase cleavage site from a cohesin subunit, RAD21, targeted to chromosomes through the N-terminal fusion of histone H2B with two fluorescent proteins, mScarlet and mNeonGreen, flanking the separase cleavage site. High separase activity at anaphase I would remove mNeonGreen from the fusion protein, while mScarlet signals remain on chromosomes. Live imaging during the metaphase I–anaphase I transition showed similar rates of loss of mNeonGreen signals on chromosomes in both *domesticus* and hybrid oocytes (Fig. 2A), implying that the separase activity is not substantially impaired in hybrid oocytes. Consistent with this observation, sister kinetochores were split in meiosis II hybrid eggs (fig. S3), indicating active separase cleaving centromeric cohesin (*31*). A limitation of this experiment is that we used a human RAD21 fragment rather than a more relevant REC8 fragment from *domesticus* and *spicilegus*. Nevertheless, the separase cleavage sites on RAD21 and REC8 are identical between *domesticus* and *spicilegus*, and therefore, it is unlikely that separase-kleisin (RAD21 and REC8) incompatibility or lower separase activity explain the observed nondisjunction phenotype.

Next, we tested the second possibility regarding cohesin overprotection by the SGO-PP2A complex. SGO2, the major SGO protein in mouse oocytes (*50*, *51*), localizes to the (peri)centromere region to protect cohesin. Since SGO2 is typically restricted to the centromere, cohesin along arms is not protected and is thus cleaved by separase at anaphase I. We analyzed the localization pattern of SGO2 in hybrid oocytes, finding that SGO2 was aberrantly localized along chromosome axes in contrast to its centromeric localization in both pure species (Fig. 2B). To test whether defective homolog separation depends on SGO2, we depleted SGO2 by RNA interference (RNAi; Fig. 2C and fig. S4). In *domesticus* oocytes, depletion of SGO2 during meiosis I caused precocious separation of sister chromatids (PSSC) due to the deprotection of cohesin (*50*, *51*). SGO2 depletion in hybrid oocytes also resulted in PSSC, indicating that defective homolog separation depends on SGO2. These results suggest that SGO2 mislocalization drives cohesin protection along arms, preventing cohesin cleavage at anaphase I and leading to aneuploid eggs (Fig. 2D).

**Fig. 3.**
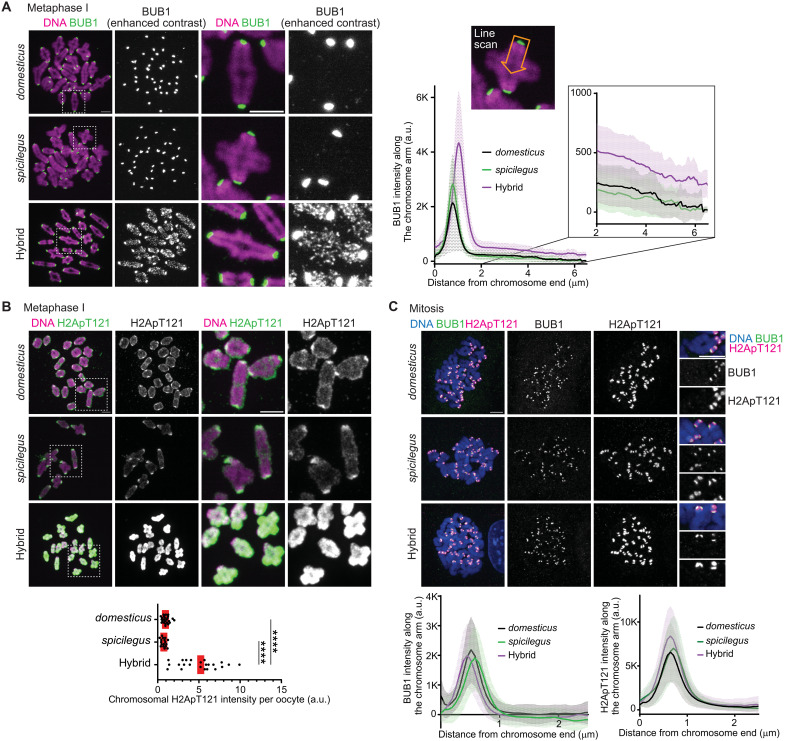
BUB1-H2ApT121 pathway is up-regulated in hybrid oocytes. (**A**) Chromosome spreads using *domesticus*, *spicilegus*, and hybrid metaphase I oocytes were stained for BUB1. Line scans show BUB1 intensities along the chromosome arm starting from the centromere. Lines indicate the mean values of BUB1 intensity along the chromosome arm; shaded regions, SD (*n* = 79, 80, and 132 chromosomes for *domesticus*, *spicilegus*, and hybrid, respectively). Enlarged area of the graph highlights the difference of BUB1 intensities along the chromosome arm. (**B**) Chromosome spreads using *domesticus*, *spicilegus*, and hybrid metaphase I oocytes were stained for H2ApT121. Graph shows chromosomal H2ApT121 intensities per oocyte (*n* = 24, 17, and 23 oocytes for *domesticus*, *spicilegus*, and hybrid); each dot in the graph represents one oocyte; red line, median; Mann-Whitney test was used for statistical analysis; ****P < 0.0001. (**C**) Granulosa cells from *domesticus*, *spicilegus*, and hybrid were fixed and stained for BUB1 and H2ApT121. Line graphs show BUB1 and H2ApT121 intensities along the chromosome arm as in (A) (*n* = 91, 121, and 142 chromosomes for *domesticus*, *spicilegus*, and hybrid). Scale bars, 5 μm.

A conserved kinetochore kinase, BUB1, phosphorylates histone H2A (H2ApT121 in mice) at the centromere region, which serves as a scaffold to recruit SGO2 in mouse and human oocytes (*34*, *52*, *53*). Since BUB1 is the upstream kinase for SGO2, we speculated that BUB1 misregulation causes ectopic localization of SGO2 in hybrid oocytes. We found that while BUB1 is enriched at the kinetochore in both pure species, BUB1 levels were increased at the kinetochore and along chromosome arms in hybrid oocytes (Fig. 3A). As a result, the H2ApT121 mark covered the entire chromosome in hybrid oocytes unlike its centromeric enrichment in pure species (Fig. 3B). We also analyzed the localization of BUB1 and H2ApT121 in mitosis, using granulosa cells (mitotically growing cells in the ovary). In both hybrid and pure species, BUB1 and H2ApT121 remained restricted to centromeres in mitosis (Fig. 3C), suggesting that hybrid incompatibility in cohesin protection is meiosis specific, consistent with viable hybrid mice.

**Fig. 4.**
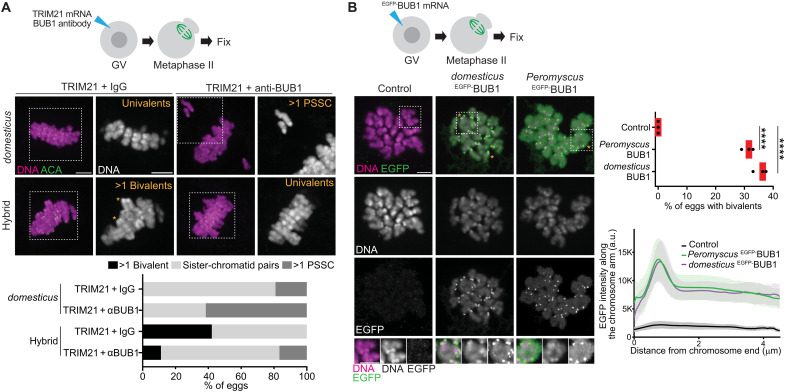
Higher BUB1 activity is sufficient to induce nondisjunction. (**A**) *domesticus* and hybrid oocytes microinjected with mCherry-TRIM21 mRNA together with immunoglobulin G (IgG) or BUB1 antibody were fixed at metaphase II and stained for ACA; asterisks, bivalents. Percentage of eggs with >1 bivalent, >1 PSSC, and sister-chromatid pairs were quantified (*n* = 21, 26, 38, and 54 eggs for *domesticus* TRIM21 + IgG, *domesticus* TRIM21 + anti-BUB1, hybrid TRIM21 + IgG, and hybrid TRIM21 + anti-BUB1, respectively). (**B**) *domesticus* oocytes expressing ^EGFP-^BUB1 derived from *domesticus* or *P. maniculatus* were fixed at metaphase II and stained for EGFP; asterisks, bivalents. Percentage of meiosis II eggs with >1 bivalent was quantified (*n* = 40, 34, and 51 oocytes for control, *domesticus* ^EGFP-^BUB1, and *Peromyscus* ^EGFP-^BUB1); each dot in the graph represents one experiment; red line, mean; unpaired two-tailed *t* test was used for statistical analysis; ****P < 0.0001. Line scans show EGFP intensities along the chromosome arm starting from the centromere. Lines indicate the mean values of EGFP intensity along the chromosome arm; shaded regions, SD (*n* = 14, 24, and 29 oocytes for control, *domesticus* ^EGFP-^BUB1, *Peromyscus* ^EGFP-^BUB1). Scale bars, 5 μm.

To directly test whether higher BUB1 levels cause cohesin protection errors in hybrid oocytes, we depleted BUB1 by Trim-Away (Fig. 4A and fig. S5). In control *domesticus* oocytes, BUB1 depletion increased the number of eggs with sister separation (i.e., PSSC), consistent with BUB1’s role in protecting cohesin (*52*). BUB1 depletion in hybrid oocytes also induced PSSC but, importantly, substantially reduced eggs with unseparated bivalents. As a complementary approach, we overexpressed BUB1 in *domesticus* oocytes to test whether higher BUB1 levels are sufficient to drive the nondisjunction phenotype. Overexpressed *domesticus* ^EGFP-^BUB1 localized on kinetochores and the arms, producing meiosis II eggs with bivalents by increasing SGO2 levels on the arms (Fig. 4B and fig. S6). Sister kinetochores were split in these bivalents (Fig. 4B, enlarged insets), mimicking the bivalents in meiosis II hybrid eggs (fig. S3). These results are consistent with the idea that the higher BUB1 activity/level in hybrid oocytes drives cohesin overprotection, leading to female sterility.

**Fig. 5.**
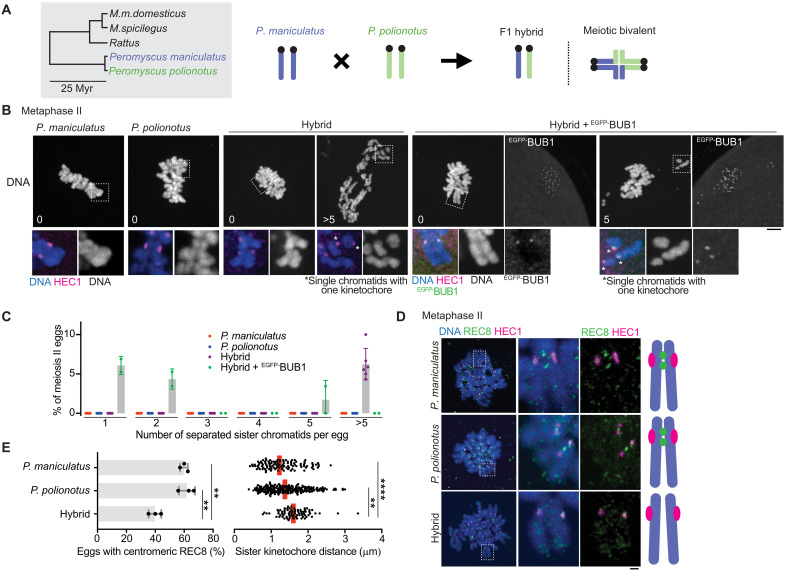
Weaker cohesin protection in *Peromyscus* hybrid oocytes. (**A**) Phylogenetic tree including *Peromyscus* and schematic of the hybrid *Peromyscus* system. (**B**) *P. maniculatus*, *P. polionotus*, and their hybrid oocytes (with or without ^EGFP-^BUB1 expression) were matured to meiosis II, fixed, and stained for HEC1. Number in the DNA images indicates the number of split sister-chromatids in the egg. (**C**) The number of the separated sister-chromatids in each meiosis II egg was quantified (*n* = 162, 171, 147, and 50 eggs for *P. maniculatus*, *P. polionotus*, hybrid, and hybrid + ^EGFP-^BUB1, respectively); each dot represents one experiment; bars and error bars represent mean and SD, respectively. (**D**) Chromosome spreads from *P. maniculatus*, *P. polionotus*, and hybrid metaphase II eggs were stained for REC8 and HEC1. (**E**) Bar graph shows the proportion of meiosis II eggs with centromeric REC8 signals in each genotype (*n* = 32, 21, and 40 eggs for *P. maniculatus*, *P. polionotus*, and the hybrid, respectively); each dot represents one experiment; bars and error bars represent mean and SD, respectively; unpaired two-tailed *t* test was used for statistical analysis. Images from (D) were used for the quantification. Scatterplot is a quantification of sister-kinetochore distance at metaphase II; images from (B) were used for the quantification; red line, median; Mann-Whitney test was used for statistical analysis; **P < 0.01 and ****P < 0.0001. Scale bars, 5 μm.

**Fig. 6.**
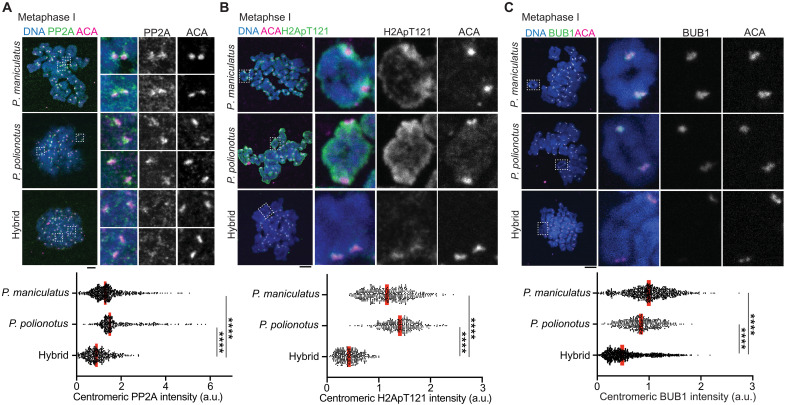
BUB1-H2ApT121 pathway is down-regulated in *Peromyscus* hybrid oocytes. (**A** to **C**) Chromosome spreads from *P. maniculatus*, *P. polionotus*, and hybrid metaphase I oocytes were stained for ACA together with PP2A (A), H2ApT121 (B), or BUB1 (C). Centromeric intensities for PP2A (*n* = 678, 403, and 448 centromeres for *P. maniculatus*, *P. polionotus*, and the hybrid, respectively), H2ApT121 (*n* = 473, 301, and 322 centromeres for *P. maniculatus*, *P. polionotus*, and the hybrid, respectively), and BUB1 (*n* = 805, 394, and 1213 centromeres for *P. maniculatus*, *P. polionotus*, and the hybrid, respectively) were quantified; each dot represents one centromere; red line, median; Mann-Whitney test was used for statistical analysis. ****P < 0.0001; scale bars, 5 μm.

Since cohesin subunits and their regulators evolve rapidly, cohesion defects may occur in other hybrids (*13*, *54*). While searching for other hybrid mouse models with cohesin protection errors, we found that hybrid female mice between *Peromyscus maniculatus* and *Peromyscus polionotus* showed substantial cohesion defects (i.e., >5 separated sister chromatids) in 5 to 10% of meiosis II eggs, while pure species showed no precocious separation (Fig. 5, A to C) (*55*). Visualizing REC8 cohesin in *Peromyscus* meiosis II eggs is technically challenging (*56*), but we found that eggs with centromeric REC8 signals were reduced in the hybrid compared to control pure species (Fig. 5, D and E). As a functional readout of reduced centromeric cohesin, we measured sister-kinetochore distance at metaphase II (Fig. 5E, right graph). Consistent with the REC8 staining result, hybrid eggs showed slightly but substantially increased sister-kinetochore distance compared to pure species. These observations imply a cohesin protection error similar to the *domesticus* x *spicilegus* hybrid but in the opposite direction, weakening protection. To test this idea, we examined the localization of PP2A, which functions with SGO2 to protect cohesin (*51*, *56*–*59*). We found that PP2A was reduced at the centromere in hybrid oocytes compared to pure species oocytes (Fig. 6A), supporting the idea that hybrid oocytes show weaker cohesin protection. Why do *Peromyscus* hybrid oocytes recruit less PP2A at their pericentromeres? We found that BUB1 kinase and H2ApT121 levels were substantially lower at the centromere in hybrid oocytes compared to control pure species oocytes (Fig. 6, B and C). Overexpressing *Peromyscus* ^EGFP-^BUB1 partially rescued cohesin protection defects (Fig. 5, B and C, no eggs with >5 separated sister chromatids), implying that BUB1 misregulation is a major cause of defective cohesin protection in *P. maniculatus* x *P. polionotus* hybrid oocytes. *Peromyscus* ^EGFP-^BUB1 overexpression also induced unseparated bivalents in *domesticus* oocytes, further confirming its functionality (Fig. 4B). *P. maniculatus* x *P. polionotus* hybrid females do not exhibit substantial fertility defects (*60*, *61*), and therefore, we believe that our cell biological approach has identified an evolving reproductive barrier in this genus.

## DISCUSSION

This work provides the cell biological evidence that cohesin protection errors can be a reproductive isolating barrier. While there are examples of hybrid incompatibility that belong to the same category (e.g., oxidative respiration and nuclear trafficking) (*1*), we think that this is an example of recurrent incompatibility in the same molecular pathway (i.e., cohesin protection) identified in hybrid animals from two genera. Future investigation may reveal the molecular basis underlying how the BUB1 activity is misregulated in each hybrid system. We detected a slightly higher BUB1 mRNA levels in the *domesticus* x *spicilegus* hybrid compared to pure species in an oocyte-specific manner (fig. S7). However, ectopic BUB1 overexpression in pure species does not fully recapitulate the robust SGO2 relocalization to the chromosome axis and multiple nondisjoined chromosomes observed in *domesticus* x *spicilegus* eggs (Figs. 1G, 2B, and 4B and fig. S6). Thus, it would be also important to examine other possible upstream regulators, including KNL1 and MPS1, which are critical to recruit BUB1 to kinetochores in pure species (*62*–*64*). MPS1 could be of particular interest since earlier work has proposed its role in directly regulating SGO2 localization independently of BUB1 (*52*).

Female fertility in humans follows an inverted U-shape, with reduced fertility in teenagers and women of advanced ages (*65*). Fertility reduction has a strong correlation with higher egg aneuploidy rates. Juvenile oocytes show higher nondisjunction rates of homologous chromosomes, similar to *domesticus* x *spicilegus* oocytes. In contrast, aged oocytes exhibit PSSC due to cohesin loss and weakened cohesin protection (*34*, *37*, *38*, *65*, *66*), resembling *P. maniculatus* x *P. polionotus* oocytes. Therefore, our study suggests that major causes of human infertility may play positive roles in mammalian speciation. Last, together with our previous work on hybrid incompatibility in chromosome condensation, misregulation of chromosome structure and dynamics may represent recurrent reproductive isolating barriers in mice (*8*).

## MATERIALS AND METHODS

### Mouse strains

Mouse strains were purchased from Envigo [Non-Swiss Albino (NSA), stock #033 corresponds to CF1, *M. m. domesticus*], the Jackson Laboratory (C57BL/6J, stock #000664, *M. m. domesticus*, PANCEVO/EiJ, stock #001384, *M. spicilegus*), RIKEN BioResource Research Center (ZBN/Ms, stock #RBRC00661, *M. spicilegus*; developed by T. Koide at Mouse Genomics Resource Laboratory), and the Peromyscus Genetic Stock Center at the University of South Carolina [*P. bairdii* (BW strain) and *P. polionotus subgriseus* (PO strain)]. CF-1 and C57BL/6J have complementary advantages as *M. m. domesticus* strains: CF-1 is an outbred strain with a substantially higher oocyte yield, and C57BL/6J is an inbred strain that efficiently produces hybrid offspring with PANCEVO/EiJ and ZBN/Ms. For most experiments, *M. m. domesticus* C57BL/6J females were crossed to *M. spicilegus* ZBN/Ms males to generate F1 hybrids. The other direction is less efficient in producing offspring, and we specifically used them in fig. S1C. C57BL/6J x PANCEVO/EiJ F1 hybrid females were used for the initial characterization of the hybrid [one of the independent experiments in Figs. 1 (D and H) and 2 (A and C)]. The mouse strain is now discontinued at the Jackson Laboratory, and we have completely switched to ZBN/Ms. *P. maniculatus* females were crossed to *P. polionotus* males to produce F1 hybrids, because the other direction does not produce viable hybrid (*67*). All animal experiments were approved by the Animal Care and Use Committee (National Institutes of Health Animal Study Proposal #H-0327) and were consistent with the National Institutes of Health guidelines.

### Mouse oocyte collection and culture

Fully grown germinal vesicle (GV)–intact prophase I oocytes were harvested from 6- to 10-week-old female mice in the M2 media (Sigma-Aldrich, catalog no. M7167) supplemented with 5 μM milrinone (Sigma-Aldrich, catalog no. 475840) to prevent meiotic resumption (*68*). After the oocyte collection, oocytes were transferred to the M16 media (Millipore, catalog no. M7292) containing 5 μM milrinone covered with paraffin oil (Nacalai, catalog no. NC1506764) and incubated at 37°C in a humidified atmosphere of 5% CO_2_ in air. To induce meiotic resumption, milrinone was washed out, and oocytes that did not undergo the nuclear envelope breakdown (NEBD) within 1.5 hours after the milrinone washout were removed from the culture. For the in situ chromosome counting assay in fig. S2, oocytes collected from 12-week to 1-year-old females were matured for 13 hours after NEBD and subsequently treated with 100 μM monastrol (Millipore, catalog no. 475879) in the organ culture dish (Falcon, catalog no. 353037) for 2 hours 15 min before the fixation (*68*). To label chromosomes during live imaging, SPY650-DNA (1:1000 dilution; Spirochrome, catalog no. SC501) was added to oocyte culture upon NEBD.

### Plasmid construction and cRNA synthesis

Coding sequences of *M. m. domesticus* and *P. maniculatus bairdii* (BW strain) BUB1 were subcloned into the plasmid in vitro transcription vector with an N-terminal enhanced green fluorescent protein (EGFP) (*69*). The plasmids were linearized with Aat II and used as DNA templates to synthetize complementary RNA (cRNA; see below). The separase sensor construct was a gift from I. M. Cheeseman (*49*). The following primers 5′GAATTAATACGACTCACTATAGGCCGGCGCCACCATGCCAGAG3′ and 5′GCCTCCAAAAAAGCCTCCTCACTACTTCTGGAATAGCTCAGAG3′ were used for polymerase chain reaction (PCR) amplification to fuse the T7 promoter sequence to the 5′ end of the sensor construct template DNA. cRNA was synthesized using the template DNA and T7 mMessage mMachine kit (Ambion, catalog no. AM1340) and purified using the MEGAclear kit (Thermo Fisher Scientific, catalog no. AM1908).

### Oocyte microinjection and electroporation

GV-intact prophase I oocytes were microinjected with ~5 pl of cRNA or antibodies in M2 containing 5 μM milrinone, using a micromanipulator TransferMan 4r and FemtoJet 4i (Eppendorf). cRNA used for microinjections was *Egfp-Bub1* (*M. m. domesticus* or *P. maniculatus bairdii* BUB1 fused with EGFP at the N terminus, 1184 and 950 ng/μl, respectively), *mCherry-Trim21* (Addgene catalog no. 105522, *M. musculus domesticus* TRIM21 fused with mCherry at the C terminus, 1500 or 3000 ng/μl for BUB1 and REC8 Trim-Away, respectively), and *hH2B-mScarlet-hRad21-mNeonGreen* [separase sensor, pNM853, human H2B fused with human Rad21 (142 to 476 amino acids) at the C terminus with the Rad21 fragment flanked by two fluorescent proteins, mScarlet and mNeonGreen, 750 ng/μl]. Following microinjections, oocytes were maintained at prophase I in M16 supplemented with 5 μM milrinone overnight to allow protein expression. To Trim-Away BUB1, GV oocytes were microinjected with *mCherry-Trim21* cRNA together with normal goat immunoglobulin G (IgG; EMD Millipore, catalog no. N102) or anti-BUB1 antibody (gift from S. S. Taylor). The oocytes were transferred to M16 supplemented with 5 μM milrinone for 2 hours to recover and then transferred to M16 for overnight maturation to metaphase II. To Trim-Away REC8, GV oocytes were microinjected with *mCherry-Trim21* cRNA. The oocytes were matured to metaphase II overnight in M16, microinjected with anti-REC8 antibody (gift from M. A. Lampson) using piezoXpert (Eppendorf), and incubated in M16 for additional 3 hours prior fixation. For SGO2 RNAi, GV oocytes were electroporated with control (Stealth RNAi siRNA Negative Control, Med GC, Invitrogen, catalog no. 12935300) and SGO2 small interfering RNA [siRNA; custom RNAi from Invitrogen, ggataaagacttcccaggaacttta (*50*)] at 200 nM using NEPA21 Super electroporator Type II (NEPA GENE). The oocytes were transferred to M16 supplemented with 5 μM milrinone, cultured for 24 hours for efficient knockdown, and transferred to M16 for overnight maturation to metaphase II before fixation.

### Immunostaining of whole oocytes and chromosome spreads

For whole-oocyte immunostaining, meiosis II eggs were fixed at metaphase II (16 hours from NEBD) in freshly prepared 2% paraformaldehyde (Electron Microscopy Sciences, catalog no. 15710) in 1× phosphate-buffered saline (PBS; Quality Biological, catalog no. 119-069-101CS) with 0.1% Triton X-100 (Millipore, catalog no. TX1568-1) for 20 min at room temperature (RT), permeabilized in 1× PBS with 0.1% Triton X-100 for 15 min at RT, placed in the blocking solution [0.3% bovine serum albumin (Fisher Bioreagents, catalog no. BP1600-100) and 0.01% Tween 20 (Thermo Fisher Scientific, catalog no. J20605-AP) in 1× PBS] overnight at 4°C, incubated for 2 hours with primary antibodies at RT, washed three times for 10 min with the blocking solution, incubated for 1 hour with secondary antibodies at RT, washed three times for 10 min in the blocking solution, and mounted on microscope slides with the Antifade Mounting Medium with 4′,6-diamidino-2-phenylindole (DAPI; Vector Laboratories, catalog no. H-1200).

For chromosome spreads, zona pellucida was removed from oocytes/eggs, and the oocytes/eggs were fixed with 1% paraformaldehyde, 0.15% Triton X-100, and 3 mM dithiothreitol (Sigma-Aldrich, catalog no. 43815) at metaphase I (7 hours from NEBD) or at metaphase II (16 hours from NEBD)

The following primary antibodies were used: rabbit anti-mouse REC8 antibody (1:500; gift from M. A. Lampson), mouse anti-human HEC1 antibody (1:100; Santa Cruz, catalog no. sc-515550), rabbit anti-mouse SGO2 antibody (1:50; gift from K.-I. Ishiguro and Y. Watanabe), CREST human autoantibody against centromere (1:100; Immunovision, catalog no. HCT-0100), sheep polyclonal anti human-BUB1 antibody, SB1.3 (1:100; gift from S. S. Taylor), rabbit anti-H2ApT120 antibody (1:2500; Active motif, catalog no. 39391), goat anti-GFP antibody (1:100; Rockland, 600-101-215M), and mouse anti-human PP2A C subunit (1:100; EMD Millipore, catalog no. 05-421-AF488). Secondary antibodies were Alexa Fluor 488–conjugated donkey anti-mouse (1:500; Invitrogen, catalog no. A21202) or donkey anti-goat (1:500; Invitrogen, catalog no. A11057), Alexa Fluor 568–conjugated goat anti-rabbit (1:500; Invitrogen, catalog no. A10042), or Alexa Fluor 647–conjugated goat anti-human (1:500; Invitrogen, catalog no. A21445).

### Granulosa cell culture and immunostaining

The procedure for isolating and culturing ovarian granulosa cells has been described previously (*70*). Briefly, after euthanizing the mice, their ovaries were collected and rinsed three times with M2 media (Sigma-Aldrich, catalog no. M7167) to remove any adherent fat tissue. The ovaries were then mechanically disrupted to release oocytes and granulosa cells. Following the collection of oocytes, the remaining granulosa cells were collected into a 15-ml tube and allowed to settle at the bottom for 5 to 10 min. The supernatant was discarded to remove blood cells, and the granulosa cells were then centrifuged at 500*g* for 5 min. The cells were washed extensively with Dulbecco’s modified Eagle’s medium (DMEM) high-glucose GlutaMAX media (Gibco, catalog no. 10566-016) supplemented with 1× antibiotic-antimycotic (Gibco, catalog no. 15240062). Cells were dispersed by pipetting, washed for two additional times, seeded on glass-bottom chamber slides (Lab-Tek, catalog no. 155411) with DMEM supplemented with 10% fetal bovine serum (Gibco, catalog no. A3160501) and 1× antibiotic-antimycotic, and cultured until they reach 50% confluency in a humidified atmosphere containing 5% CO_2_ at 37°C. After 24 hours, the medium was replaced with fresh media of the same type to continue the primary culture. Mitotic cells were enriched by adding 1 μM nocodazole (Sigma-Aldrich, catalog no. 487929-10MG-M) to the medium, and the cells were cultured for 10 hours before proceeding to immunostaining (see above).

### Quantitative real-time PCR

Gene expression levels were quantified by quantitative real-time PCR (RT-qPCR). EGFP mRNA was added to TRIzol (Invitrogen, catalog no. 15596026) as an exogenous mRNA control for the RT-qPCR to normalize BUB1 mRNA levels. Total RNA was extracted from 20 to 30 full-grown GV oocytes or from ovarian granulosa cells using the TRIzol-EGFP mRNA mixture followed by chloroform (MP Biomedicals, catalog no. 193814). The mixture was centrifuged at 12,000*g* for 15 min at 4°C. The upper aqueous solution was collected and mixed with 10 μg of GlycoBlue Coprecipitant (Invitrogen, catalog no. AM9515) and 2-propanol (J.T.Baker, catalog no. 9084-01) and then centrifuged at 12,000*g* for 10 min at 4°C. The RNA pellet was then washed with 75% ethanol followed by centrifugation at 7500*g* for 5 min. The pellet was air dried and eluted in 8 and 10 μl of nuclease-free water, for oocytes and granulosa cells, respectively. The cDNA was prepared by reverse transcription of total RNA volume (8 μl) from oocytes or ~1 μg of total RNA from granulosa cells, with Superscript III First Strand Synthesis System (Invitrogen, catalog no. 18080051) using oligo dT primers. Quantification was performed by the 2^−ΔΔ*C*^_T_ method (*71*) using EGFP as the reference. RT-qPCR was performed using iTaq Universal SYBR Green Supermix (Bio-Rad, catalog no. 1725121) in a QuantStudio 3 Real-Time PCR System (Thermo Fisher Scientific) and LightCycler 96 (Roche) in 96-well PCR plates sealed with microseal (Bio-Rad, catalog no. MSC-1001). BUB1 and reference genes were analyzed at the same time in triplicates of six and three independent samples of oocytes and granulosa cells, respectively. The primers used to amplify the cDNA were as follows: BUB1, 5′CATGAGCAGTGGGTTAGTGAAGAC 3′ (forward) and 5′TTCTCAGAAGCAGGAAGGTCCTTG 3′ (reverse); EGFP, 5′AAGGGCATCGACTTCAAGG 3′ (forward) and 5′TGCTTGTCGGCCATGATATAG 3′ (reverse).

### Confocal microscopy

Fixed eggs, chromosome spreads, and granulosa cells were imaged with a microscope (Eclipse Ti; Nikon) equipped with 100×/1.40 numerical aperture (NA) oil-immersion objective lens, CSU-W1 spinning disk confocal scanner (Yokogawa), ORCA Fusion Digital complementary metal-oxide semiconductor (CMOS) camera (Hamamatsu Photonics), and 405-, 488-, 561-, and 640-nm laser lines controlled by the NIS-Elements imaging software (Nikon). Confocal images were acquired as Z-stacks at 0.3-μm intervals. For live imaging, oocytes were placed into 3-μl drops of M16 covered with paraffin oil in a glass-bottom tissue culture dish (FluoroDish, catalog no. FD35-100) in a stage top incubator (Tokai Hit) to maintain 37°C and 5% CO_2_. Time-lapse images were collected with a microscope (Eclipse Ti2-E; Nikon) equipped with the 20×/0.75 NA objective (Figs. 1D and 2A and fig. S1, A and B), CSU-W1 spinning disk confocal scanner (Yokogawa), ORCA Fusion Digital CMOS camera (Hamamatsu Photonics), and 405-, 488-, 561-, and 640-nm laser lines controlled by the NIS-Elements imaging software (Nikon). Confocal images were collected as Z-stacks at 3-μm intervals to visualize all the chromosomes. Images are displayed as maximum intensity Z-projections in the figures.

### Image analysis

Fiji/ImageJ (National Institutes of Health) was used to analyze all the images. In general, optical slices containing chromosomes were added to produce a sum intensity Z-projection for pixel intensity quantifications. For line scans of SGO2, BUB1, and H2ApT121 signal intensities (Figs. 2B; 3, A and C; and 4B), lines (width: 11 pixels) were drawn from the centromere toward the chromosome arm, and signal intensities were averaged over multiple chromosomes after subtracting background signals, obtained near the chromosome. To quantify chromosomal H2ApT121 signal intensities in oocytes (Fig. 3B), masking images were created using DAPI staining images to specifically measure the signal intensities on the chromosome. The signal intensity was integrated over each slice after subtracting the background, obtained near the chromosomes. For the quantification of the separase sensor cleavage (Fig. 2A), masking images were created using mScarlet images to measure signal intensities of mNeonGreen and mScarlet on the chromosomes from metaphase I to anaphase I. Signal intensities were integrated over each slice after subtracting the background, obtained near the chromosomes. The mNeonGreen/mScarlet ratio was calculated for each time point. For the in situ chromosome counting assay (fig. S2), the number of chromosomes was counted in metaphase II eggs using the DAPI and anticentromere antibody (ACA) signals. To specifically quantify centromeric signal intensities [PP2A, BUB1, and H2ApT121 in Fig. 6 (A to C)], ellipses were delineated around the centromere (based on the ACA staining) on each chromosome. Signal intensities were then quantified within each ellipse after the background signal subtraction. To classify bivalents, sister-chromatids, and PSSC (Figs. 1H, 2C, and 4A), we primarily used DAPI signals and focused on the centromere position (the DAPI-dense region) relative to the chromosome arms: Bivalents have centromeres positioned at both ends of the chromosome with the arms located in between them, and sister-chromatids have centromeres positioned in the middle with the arms sprayed out. Isolated single chromatids with a single centromere were classified as PSSC. ACA signals at the centromeres were used to aid the classification.

### Statistics and reproducibility

Data points were pooled from two independent experiments except for fig. S1C, which has one experiment due to the difficulty of producing the hybrid using *M. spicilegus* females, Figs. 1D, 4B, and 5E and figs. S1A, S2, and S7 granulosa cell RT-qPCR, which have three biological replicates, and fig. S7 oocyte RT-qPCR, which has six biological replicates. Statistical analyses were performed using Microsoft Excel and GraphPad Prism 10. Scatterplots and line and bar graphs were created with GraphPad Prism 10. Mann-Whitney test and unpaired two-tailed *t* test were used for statistical analysis unless specified in the figure legend, and a value of *P* < 0.05 was considered significant.

## References

[1] N. A. Johnson, Hybrid incompatibility genes: Remnants of a genomic battlefield? Trends Genet. 26, 317–325 (2010). 20621759 10.1016/j.tig.2010.04.005

[2] N. Phadnis, H. A. Orr, A single gene causes both male sterility and segregation distortion in *Drosophila* hybrids. Science 323, 376–379 (2009). 19074311 10.1126/science.1163934 PMC2628965

[3] M. Kitaoka, O. K. Smith, A. F. Straight, R. Heald, Molecular conflicts disrupting centromere maintenance contribute to *Xenopus* hybrid inviability. Curr. Biol. 32, 3939–3951.e6 (2022). 35973429 10.1016/j.cub.2022.07.037 PMC9529917

[4] J. Bloom, R. Green, A. Desai, K. Oegema, S. A. Rifkin, Hybrid incompatibility emerges at the one-cell stage in interspecies *Caenorhabditis* embryos. bioRxiv 619171 [Preprint] 2024.10.19.619171 (2024). 10.1101/2024.10.19.619171. PMC12258963 40602404

[5] T. A. Suzuki, M. W. Nachman, Speciation and reduced hybrid female fertility in house mice. Evolution 69, 2468–2481 (2015). 26299202 10.1111/evo.12747 PMC4573315

[6] O. Mihola, Z. Trachtulec, C. Vlcek, J. C. Schimenti, J. Forejt, A mouse speciation gene encodes a meiotic histone H3 methyltransferase. Science 323, 373–375 (2009). 19074312 10.1126/science.1163601

[7] N. J. Brideau, H. A. Flores, J. Wang, S. Maheshwari, X. Wang, D. A. Barbash, Two Dobzhansky-Muller genes interact to cause hybrid lethality in *Drosophila*. Science 314, 1292–1295 (2006). 17124320 10.1126/science.1133953

[8] W. El Yakoubi, T. Akera, Condensin dysfunction is a reproductive isolating barrier in mice. Nature 623, 347–355 (2023). 37914934 10.1038/s41586-023-06700-6 PMC11379054

[9] M. Sodek, K. Kovacovicova, M. Anger, True nondisjunction of whole bivalents in oocytes with attachment and congression defects. Cytogenet. Genome Res. 151, 10–17 (2017). 28278497 10.1159/000458513

[10] K. E. Koehler, S. E. Schrump, J. P. Cherry, T. J. Hassold, P. A. Hunt, Near-human aneuploidy levels in female mice with homeologous chromosomes. Curr. Biol. 16, R579–R580 (2006). 16890511 10.1016/j.cub.2006.07.018

[11] T. Hirano, Chromosome dynamics during mitosis. Cold Spring Harb. Perspect. Biol. 7, 1–14 (2015). 10.1101/cshperspect.a015792 PMC4448609 25722466

[12] I. F. Davidson, J.-M. Peters, Genome folding through loop extrusion by SMC complexes. Nat. Rev. Mol. Cell Biol. 22, 445–464 (2021). 33767413 10.1038/s41580-021-00349-7

[13] T. D. King, C. J. Leonard, J. C. Cooper, S. Nguyen, E. F. Joyce, N. Phadnis, Recurrent losses and rapid evolution of the condensin II complex in insects. Mol. Biol. Evol. 36, 2195–2204 (2019). 31270536 10.1093/molbev/msz140 PMC6759200

[14] W. Tong, H. Hoekstra, Mus spicilegus. Curr. Biol. 22, R858–R859 (2012). 23098587 10.1016/j.cub.2012.08.054

[15] J. P. F. Bonhomme, S. Catalan, P. Gerasimov, P. Orsini, L. Thaler, Le complexe d’espèces du genre Mus en Europe Centrale et Orientale I. Génétique. Mamm. Biol. 48, 78–85 (1982).

[16] B. V. E. Sokolov, E. V. Kotenkova, A. G. Michailenko, Mus spicilegus. J. Mammal. 1–6 (1998).

[17] M. B. Couger, L. Arévalo, P. Campbell, A high quality genome for *Mus Spicilegus*, a close relative of house mice with unique social and ecological adaptations. G3 (Bethesda) 8, 2145–2152 (2018). 29794166 10.1534/g3.118.200318 PMC6027863

[18] I. Lam, S. Keeney, Mechanism and regulation of meiotic recombination initiation. Cold Spring Harb. Perspect. Biol. 7, a016634 (2015). 10.1101/cshperspect.a016634 PMC4292169 25324213

[19] J. L. Gerton, R. S. Hawley, Homologous chromosome interactions in meiosis: Diversity amidst conservation. Nat. Rev. Genet. 6, 477–487 (2005). 15931171 10.1038/nrg1614

[20] O. Rog, A. F. Dernburg, Chromosome pairing and synapsis during *Caenorhabditis elegans* meiosis. Curr. Opin. Cell Biol. 25, 349–356 (2013). 23578368 10.1016/j.ceb.2013.03.003 PMC3694717

[21] C. Grey, B. de Massy, Chromosome organization in early meiotic prophase. Front. Cell Dev. Biol. 9, 688878 (2021). 34150782 10.3389/fcell.2021.688878 PMC8209517

[22] A. P. Dyban, V. S. Baranov, *Cytogenetics of Mammalian Embryonic Development* (Oxford Univ. Press, 1987).

[23] E. M. Torres, B. R. Williams, A. Amon, Aneuploidy: Cells losing their balance. Genetics 179, 737–746 (2008). 18558649 10.1534/genetics.108.090878 PMC2429870

[24] K. Ishiguro, The cohesin complex in mammalian meiosis. Genes Cell 24, 6–30 (2019). 10.1111/gtc.12652 PMC7379579 30479058

[25] I. Onn, J. M. Heidinger-Pauli, V. Guacci, E. Unal, D. E. Koshland, Sister chromatid cohesion: A simple concept with a complex reality. Annu. Rev. Cell Dev. Biol. 24, 105–129 (2008). 18616427 10.1146/annurev.cellbio.24.110707.175350

[26] S. Yatskevich, J. Rhodes, K. Nasmyth, Organization of chromosomal DNA by SMC complexes. Annu. Rev. Genet. 53, 445–482 (2019). 31577909 10.1146/annurev-genet-112618-043633

[27] T. L. Higashi, F. Uhlmann, SMC complexes: Lifting the lid on loop extrusion. Curr. Opin. Cell Biol. 74, 13–22 (2022). 35016058 10.1016/j.ceb.2021.12.003 PMC9089308

[28] Y. Watanabe, Geometry and force behind kinetochore orientation: Lessons from meiosis. Nat. Rev. Mol. Biol. 13, 370–382 (2012). 10.1038/nrm3349 22588367

[29] A. L. Marston, Shugoshins: Tension-sensitive pericentromeric adaptors safeguarding chromosome segregation. Mol. Cell. Biol. 35, 634–648 (2015). 25452306 10.1128/MCB.01176-14 PMC4301718

[30] V. Mengoli, K. Jonak, O. Lyzak, M. Lamb, L. M. Lister, C. Lodge, J. Rojas, I. Zagoriy, M. Herbert, W. Zachariae, Deprotection of centromeric cohesin at meiosis II requires APC/C activity but not kinetochore tension. EMBO J. 40, e106812 (2021). 33644894 10.15252/embj.2020106812 PMC8013787

[31] Y. Gryaznova, L. Keating, S. A. Touati, D. Cladière, W. El Yakoubi, E. Buffin, K. Wassmann, Kinetochore individualization in meiosis I is required for centromeric cohesin removal in meiosis II. EMBO J. 40, e106797 (2021). 33644892 10.15252/embj.2020106797 PMC8013791

[32] J. Kim, K. Ishiguro, A. Nambu, B. Akiyoshi, S. Yokobayashi, A. Kagami, T. Ishiguro, A. M. Pendas, N. Takeda, Y. Sakakibara, T. S. Kitajima, Y. Tanno, T. Sakuno, Y. Watanabe, Meikin is a conserved regulator of meiosis-I-specific kinetochore function. Nature 517, 466–471 (2015). 25533956 10.1038/nature14097

[33] S. Ogushi, A. Rattani, J. Godwin, J. Metson, L. Schermelleh, K. Nasmyth, Loss of sister kinetochore co-orientation and peri-centromeric cohesin protection after meiosis I depends on cleavage of centromeric REC8. Dev. Cell 56, 3100–3114.e4 (2021). 34758289 10.1016/j.devcel.2021.10.017 PMC8629431

[34] B. P. Mihalas, G. H. Pieper, M. Aboelenain, L. Munro, V. Srsen, C. E. Currie, D. A. Kelly, G. M. Hartshorne, E. E. Telfer, A. D. McAinsh, R. A. Anderson, A. L. Marston, Age-dependent loss of cohesion protection in human oocytes. Curr. Biol. 34, 117–131.e5 (2024). 38134935 10.1016/j.cub.2023.11.061 PMC7617652

[35] D. Clift, W. A. McEwan, L. I. Labzin, V. Konieczny, B. Mogessie, L. C. James, M. Schuh, A method for the acute and rapid degradation of endogenous proteins. Cell 171, 1692–1706.e18 (2017). 29153837 10.1016/j.cell.2017.10.033 PMC5733393

[36] S. Dunkley, B. Mogessie, Actin limits egg aneuploidies associated with female reproductive aging. Sci. Adv. 9, eadc9161 (2023). 36662854 10.1126/sciadv.adc9161 PMC9858517

[37] L. M. Lister, A. Kouznetsova, L. A. Hyslop, D. Kalleas, S. L. Pace, J. C. Barel, A. Nathan, V. Floros, C. Adelfalk, Y. Watanabe, R. Jessberger, T. B. Kirkwood, C. Höög, M. Herbert, Age-related meiotic segregation errors in mammalian oocytes are preceded by depletion of cohesin and Sgo2. Curr. Biol. 20, 1511–1521 (2010). 20817533 10.1016/j.cub.2010.08.023

[38] T. Chiang, F. E. Duncan, K. Schindler, R. M. Schultz, M. A. Lampson, Evidence that weakened centromere cohesion is a leading cause of age-related aneuploidy in oocytes. Curr. Biol. 20, 1522–1528 (2010). 20817534 10.1016/j.cub.2010.06.069 PMC2939204

[39] M. Tsutsumi, R. Fujiwara, H. Nishizawa, M. Ito, H. Kogo, H. Inagaki, T. Ohye, T. Kato, T. Fujii, H. Kurahashi, Age-related decrease of meiotic cohesins in human oocytes. PLOS ONE 9, e96710 (2014). 24806359 10.1371/journal.pone.0096710 PMC4013030

[40] E. Nikalayevich, S. El Jailani, A. Dupré, D. Cladière, Y. Gryaznova, C. Fosse, E. Buffin, S. A. Touati, K. Wassmann, Aurora B/C-dependent phosphorylation promotes Rec8 cleavage in mammalian oocytes. Curr. Biol. 32, 2281–2290.e4 (2022). 35385691 10.1016/j.cub.2022.03.041

[41] V. L. Katis, J. J. Lipp, R. Imre, A. Bogdanova, E. Okaz, B. Habermann, K. Mechtler, K. Nasmyth, W. Zachariae, Rec8 phosphorylation by casein kinase 1 and Cdc7-Dbf4 kinase regulates cohesin cleavage by separase during meiosis. Dev. Cell 18, 397–409 (2010). 20230747 10.1016/j.devcel.2010.01.014 PMC2994640

[42] T. Ishiguro, K. Tanaka, T. Sakuno, Y. Watanabe, Shugoshin-PP2A counteracts casein-kinase-1-dependent cleavage of Rec8 by separase. Nat. Cell Biol. 12, 500–506 (2010). 20383139 10.1038/ncb2052

[43] A. L. Marston, W.-H. Tham, H. Shah, A. Amon, A genome-wide screen identifies genes required for centromeric cohesion. Science 303, 1367–1370 (2004). 14752166 10.1126/science.1094220

[44] T. S. Kitajima, S. A. Kawashima, Y. Watanabe, The conserved kinetochore protein shugoshin protects centromeric cohesion during meiosis. Nature 427, 510–517 (2004). 14730319 10.1038/nature02312

[45] V. L. Katis, M. Galova, K. P. Rabitsch, J. Gregan, K. Nasmyth, Maintenance of cohesin at centromeres after meiosis I in budding yeast requires a kinetochore-associated protein related to MEI-S332. Curr. Biol. 14, 560–572 (2004). 15062096 10.1016/j.cub.2004.03.001

[46] N. R. Kudo, K. Wassmann, M. Anger, M. Schuh, K. G. Wirth, H. Xu, W. Helmhart, H. Kudo, M. Mckay, B. Maro, J. Ellenberg, P. de Boer, K. Nasmyth, Resolution of chiasmata in oocytes requires separase-mediated proteolysis. Cell 126, 135–146 (2006). 16839882 10.1016/j.cell.2006.05.033

[47] C. Thomas, B. Wetherall, M. D. Levasseur, R. J. Harris, S. T. Kerridge, J. M. G. Higgins, O. R. Davies, S. Madgwick, A prometaphase mechanism of securin destruction is essential for meiotic progression in mouse oocytes. Nat. Commun. 12, 4322 (2021). 34262048 10.1038/s41467-021-24554-2 PMC8280194

[48] N. Shindo, K. Kumada, T. Hirota, Separase sensor reveals dual roles for separase coordinating cohesin cleavage and cdk1 inhibition. Dev. Cell 23, 112–123 (2012). 22814604 10.1016/j.devcel.2012.06.015

[49] N. K. Maier, J. Ma, M. A. Lampson, I. M. Cheeseman, Separase cleaves the kinetochore protein Meikin at the meiosis I/II transition. Dev. Cell 56, 2192–2206.e8 (2021). 34331869 10.1016/j.devcel.2021.06.019 PMC8355204

[50] J. Lee, T. S. Kitajima, Y. Tanno, K. Yoshida, T. Morita, T. Miyano, M. Miyake, Y. Watanabe, Unified mode of centromeric protection by shugoshin in mammalian oocytes and somatic cells. Nat. Cell Biol. 10, 42–52 (2008). 18084284 10.1038/ncb1667

[51] A. Rattani, M. Wolna, M. Ploquin, W. Helmhart, S. Morrone, B. Mayer, J. Godwin, W. Xu, O. Stemmann, A. Pendas, K. Nasmyth, Sgol2 provides a regulatory platform that coordinates essential cell cycle processes during meiosis I in oocytes. eLife 2, e01133 (2013). 24192037 10.7554/eLife.01133 PMC3816256

[52] W. El Yakoubi, E. Buffin, D. Cladière, Y. Gryaznova, I. Berenguer, S. A. Touati, R. Gómez, J. A. Suja, J. M. van Deursen, K. Wassmann, Mps1 kinase-dependent Sgo2 centromere localisation mediates cohesin protection in mouse oocyte meiosis I. Nat. Commun. 8, 694 (2017). 28947820 10.1038/s41467-017-00774-3 PMC5612927

[53] S. A. Kawashima, Y. Yamagishi, T. Honda, K. Ishiguro, Y. Watanabe, Phosphorylation of H2A by Bub1 prevents chromosomal instability through localizing shugoshin. Science 327, 172–177 (2010). 19965387 10.1126/science.1180189

[54] T. Kumon, J. Ma, R. B. Akins, D. Stefanik, C. E. Nordgren, J. Kim, M. T. Levine, M. A. Lampson, Parallel pathways for recruiting effector proteins determine centromere drive and suppression. Cell 184, 4904–4918.e11 (2021). 34433012 10.1016/j.cell.2021.07.037 PMC8448984

[55] N. L. Bedford, H. E. Hoekstra, Peromyscus mice as a model for studying natural variation. eLife 4, e06813 (2015). 26083802 10.7554/eLife.06813 PMC4470249

[56] B. Pan, M. Bruno, T. S. Macfarlan, T. Akera, Meiosis-specific distal cohesion site decoupled from the kinetochore. Nat. Commun. 16, 2116 (2025). 40032846 10.1038/s41467-025-57438-w PMC11876576

[57] T. S. Kitajima, T. Sakuno, K. Ishiguro, S. Iemura, T. Natsume, S. A. Kawashima, Y. Watanabe, Shugoshin collaborates with protein phosphatase 2A to protect cohesin. Nature 441, 46–52 (2006). 16541025 10.1038/nature04663

[58] C. G. Riedel, V. L. Katis, Y. Katou, S. Mori, T. Itoh, W. Helmhart, M. Gálová, M. Petronczki, J. Gregan, B. Cetin, I. Mudrak, E. Ogris, K. Mechtler, L. Pelletier, F. Buchholz, K. Shirahige, K. Nasmyth, Protein phosphatase 2A protects centromeric sister chromatid cohesion during meiosis I. Nature 441, 53–61 (2006). 16541024 10.1038/nature04664

[59] R. C. C. Hengeveld, M. J. M. Vromans, M. Vleugel, M. A. Hadders, S. M. A. Lens, Inner centromere localization of the CPC maintains centromere cohesion and allows mitotic checkpoint silencing. Nat. Commun. 8, 15542 (2017). 28561035 10.1038/ncomms15542 PMC5460030

[60] L. R. Dice, Fertility relationships between some of the species and subspecies of mice in the genus peromyscus. J. Mammal. 10, 116–124 (1929).

[61] W. D. Dawson, Fertility and size inheritance in a *peromyscus* species cross. Evolution 19, 44–55 (1965).

[62] Y. Yamagishi, C.-H. Yang, Y. Tanno, Y. Watanabe, MPS1/Mph1 phosphorylates the kinetochore protein KNL1/Spc7 to recruit SAC components. Nat. Cell Biol. 14, 746–752 (2012). 22660415 10.1038/ncb2515

[63] L. A. Shepperd, J. C. Meadows, A. M. Sochaj, T. C. Lancaster, J. Zou, G. J. Buttrick, J. Rappsilber, K. G. Hardwick, J. B. A. Millar, Phosphodependent recruitment of Bub1 and Bub3 to Spc7/KNL1 by Mph1 kinase maintains the spindle checkpoint. Curr. Biol. 22, 891–899 (2012). 22521786 10.1016/j.cub.2012.03.051 PMC3780767

[64] N. London, S. Ceto, J. A. Ranish, S. Biggins, Phosphoregulation of Spc105 by Mps1 and PP1 regulates Bub1 localization to kinetochores. Curr. Biol. 22, 900–906 (2012). 22521787 10.1016/j.cub.2012.03.052 PMC3723133

[65] J. R. Gruhn, A. P. Zielinska, V. Shukla, R. Blanshard, A. Capalbo, D. Cimadomo, D. Nikiforov, A. C.-H. Chan, L. J. Newnham, I. Vogel, C. Scarica, M. Krapchev, D. Taylor, S. G. Kristensen, J. Cheng, E. Ernst, A.-M. B. Bjørn, L. B. Colmorn, M. Blayney, K. Elder, J. Liss, G. Hartshorne, M. L. Groendahl, L. Rienzi, F. Ubaldi, R. McCoy, K. Lukaszuk, C. Y. Andersen, M. Schuh, E. R. Hoffmann, Chromosome errors in human eggs shape natural fertility over reproductive life span. Science 365, 1466–1469 (2019). 31604276 10.1126/science.aav7321 PMC7212007

[66] F. E. Duncan, J. E. Hornick, M. A. Lampson, R. M. Schultz, L. D. Shea, T. K. Woodruff, Chromosome cohesion decreases in human eggs with advanced maternal age. Aging Cell 11, 1121–1124 (2012). 22823533 10.1111/j.1474-9726.2012.00866.x PMC3491123

[67] P. B. Vrana, X. J. Guan, R. S. Ingram, S. M. Tilghman, Genomic imprinting is disrupted in interspecific Peromyscus hybrids. Nat. Genet. 20, 362–365 (1998). 9843208 10.1038/3833

[68] P. Stein, K. Schindler, Mouse oocyte microinjection, maturation and ploidy assessment. J. Vis. Exp. 2851 (2011). 21808228 10.3791/2851 PMC3346305

[69] H. Igarashi, J. G. Knott, R. M. Schultz, C. J. Williams, Alterations of PLCbeta1 in mouse eggs change calcium oscillatory behavior following fertilization. Dev. Biol. 312, 321–330 (2007). 17961538 10.1016/j.ydbio.2007.09.028 PMC2170533

[70] J. J. Eppig, K. Wigglesworth, F. Pendola, Y. Hirao, Murine oocytes suppress expression of luteinizing hormone receptor messenger ribonucleic acid by granulosa cells. Biol. Reprod. 56, 976–984 (1997). 9096881 10.1095/biolreprod56.4.976

[71] K. J. Livak, T. D. Schmittgen, Analysis of relative gene expression data using real-time quantitative PCR and the 2^−ΔΔC^_T_ method. Methods 25, 402–408 (2001). 11846609 10.1006/meth.2001.1262

