# Supplementary material for: Hybrid female sterility due to cohesin protection errors in mouse oocytes
Source: Sci Adv. 2026 Feb 4;12(6):eadx9729. doi: 10.1126/sciadv.adx9729 (PMC12871464; doi:10.1126/sciadv.adx9729)
Supplement: Supplementary file 1 — Figs. S1 to S7 Legends for movies S1 to S3 [file sciadv.adx9729_sm.pdf]

Science Advances
AAAS

Supplementary Materials for

Hybrid female sterility due to cohesin protection errors in mouse oocytes

Warif El Yakoubi *et al.*

Corresponding author: Takashi Akera, takashi.akera@nih.gov

Sci. Adv. **12**, eadx9729 (2026)
DOI: 10.1126/sciadv.adx9729

The PDF file includes:

Figs. S1 to S7
Legends for movies S1 to S3

Other Supplementary Material for this manuscript includes the following:

Movies S1 to S3

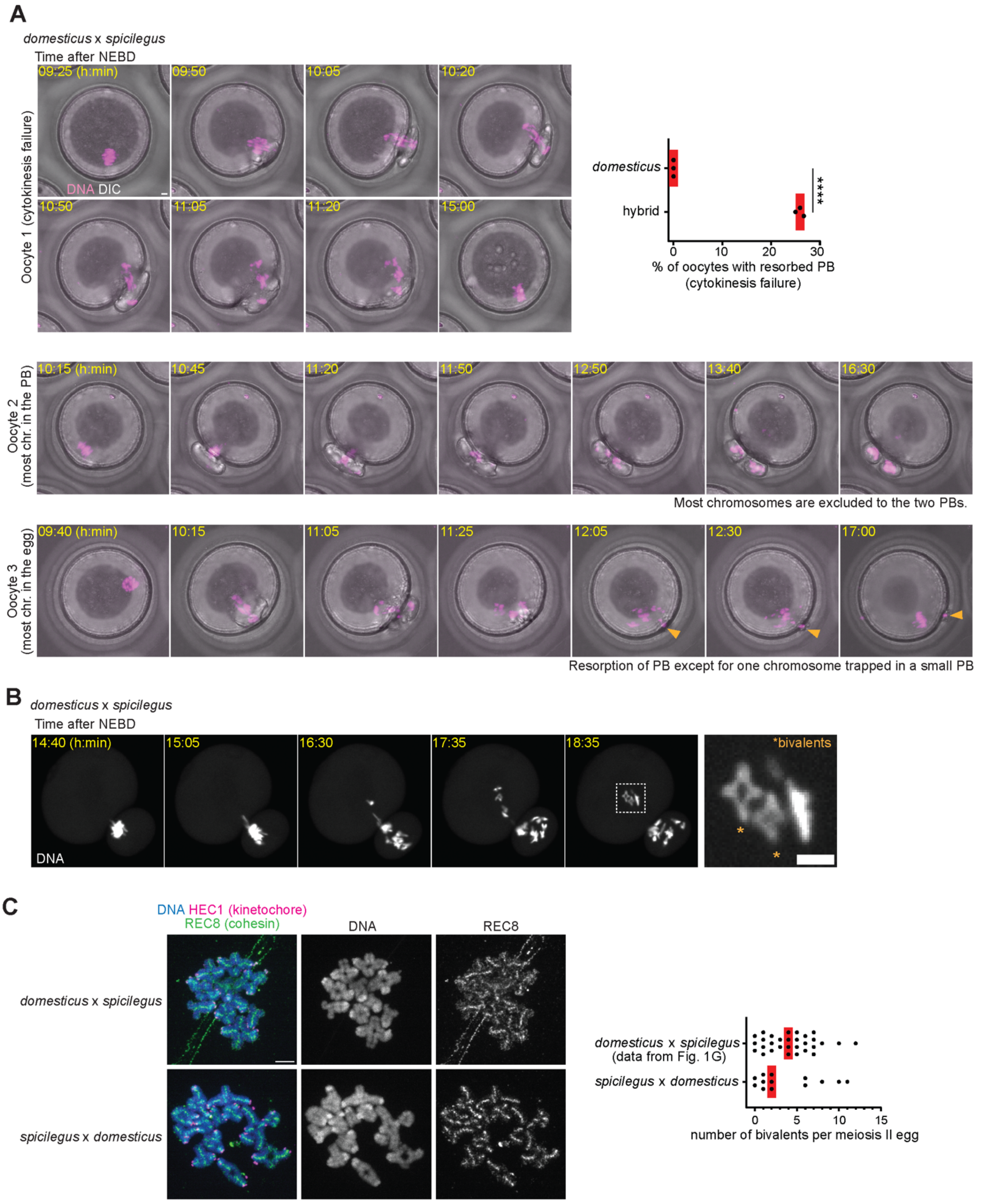

A
domesticus x spicilegus
Time after NEBD
09:25 (h:min)
09:50
10:05
10:20
DNA DIC
10:50
11:05
11:20
15:00
Oocyte 1 (cytokinesis failure)
domesticus
hybrid
****
0
10
20
30
% of oocytes with resorbed PB (cytokinesis failure)
Oocyte 2 (most chr. in the PB)
10:15 (h:min)
10:45
11:20
11:50
12:50
13:40
16:30
Most chromosomes are excluded to the two PBs.
Oocyte 3 (most chr. in the egg)
09:40 (h:min)
10:15
11:05
11:25
12:05
12:30
17:00
Resorption of PB except for one chromosome trapped in a small PB
B
domesticus x spicilegus
Time after NEBD
14:40 (h:min)
15:05
16:30
17:35
18:35
*bivalents
DNA
C
DNA HEC1 (kinetochore)
REC8 (cohesin)
DNA
REC8
domesticus x spicilegus
spicilegus x domesticus
domesticus x spicilegus (data from Fig. 1G)
spicilegus x domesticus
0
5
10
15
number of bivalents per meiosis II egg

fig. S1. Mis-segregation in hybrid oocytes is associated with cytokinetic failures. (**A**) Images from Fig. 1D were analyzed to quantify the proportion of oocytes with cytokinetic failure (Oocyte 1); red line, mean; unpaired two-tailed t test was used for statistical analysis; *****P* <0.0001. Oocyte 2 and 3 are examples of mis-segregation leading to various polar body sizes. (**B**) Examples of nondisjoined bivalent chromosomes captured by live-imaging. The images are from the time-lapse imaging dataset from Fig. 2A. (**C**) Chromosome spreads were performed at metaphase II using hybrid oocytes derived from both cross directions (i.e., *domesticus* x *spicilegus* and *spicilegus* x *domesticus*) and stained for HEC1 and REC8. Graph shows the quantification of the number of bivalents per egg (n = 30 and 13 meiosis II eggs for *domesticus* x *spicilegus* and *spicilegus* x *domesticus*, respectively); note that the data for *domesticus* x *spicilegus* is from Fig. 1G; each dot represents a single egg; red line, median.

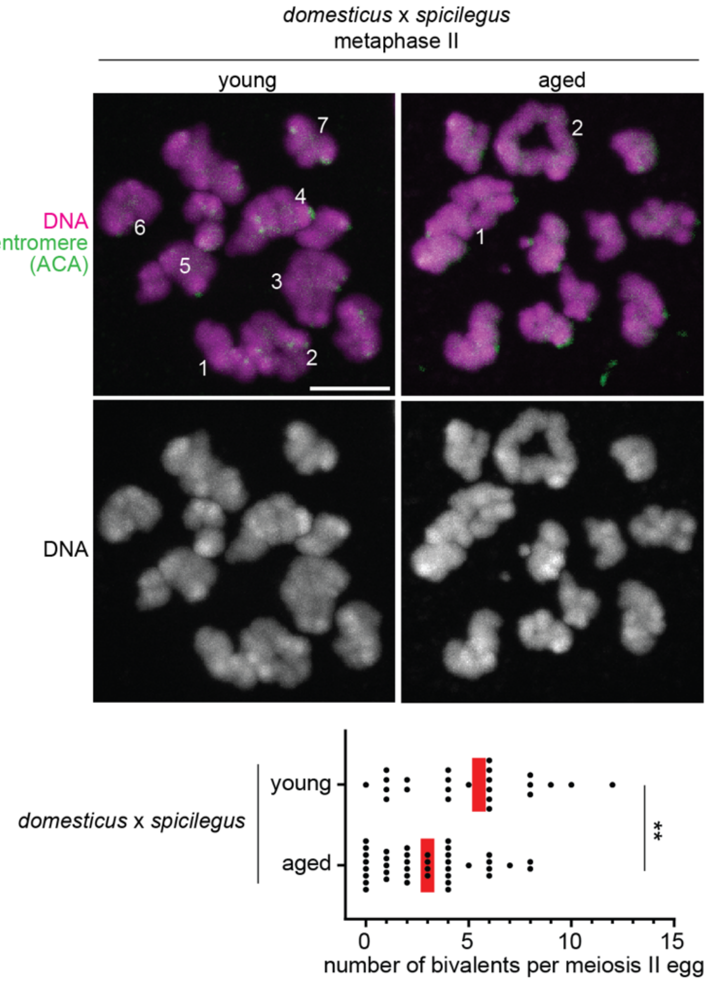

fig. S2. Hybrid meiosis II eggs from aged female mice carry fewer bivalents. Hybrid oocytes from young (3-month old) and aged (12-month old) mice were matured and fixed at metaphase II and stained for ACA (centromere). Graph quantifies the number of bivalents in each meiosis II egg (n = 21 and 36 eggs from young ang aged mice, respectively); each dot in the graph represents a single egg; red line, median; Mann-Whitney test was used for statistical analysis; $**P < 0.01$.

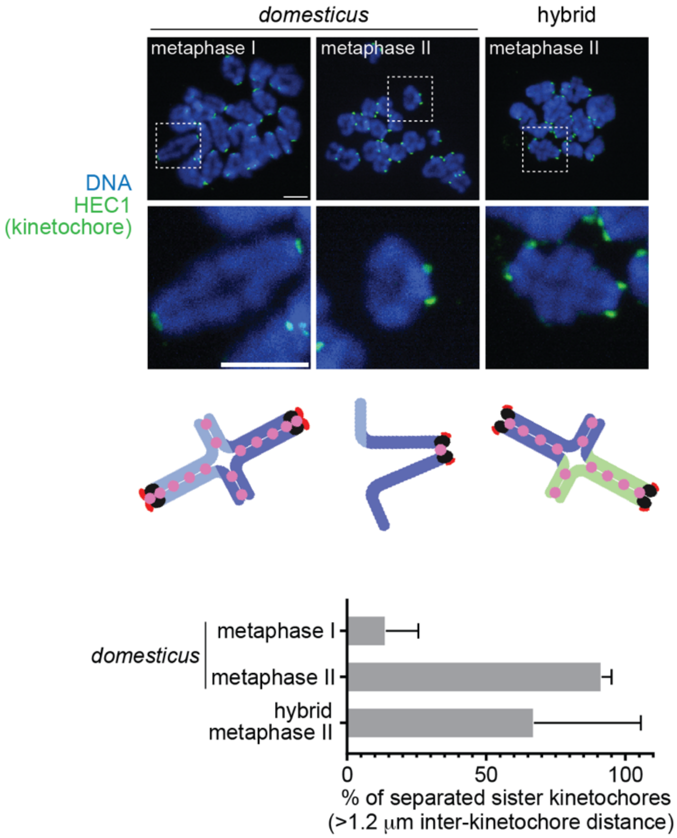

fig. S3. Sister kinetochores are normally split in unseparated bivalents. Chromosome spreads were performed at metaphase I (*domesticus*) and metaphase II (*domesticus* and the *domesticus* x *spicilegus* hybrid) and stained for HEC1. Graph shows the quantification of the percentage of chromosomes with separated sister-kinetochores (i.e., inter-kinetochore distance larger than 1.2 μm) (n = 313, 184, and 221 chromosomes for *domesticus* metaphase I, *domesticus* metaphase II, and hybrid metaphase II; bars, mean values; error bars, standard deviation for two independent experiments). Schematics were created in BioRender. El Yakoubi, W. (2025) https://BioRender.com/wa90bys.

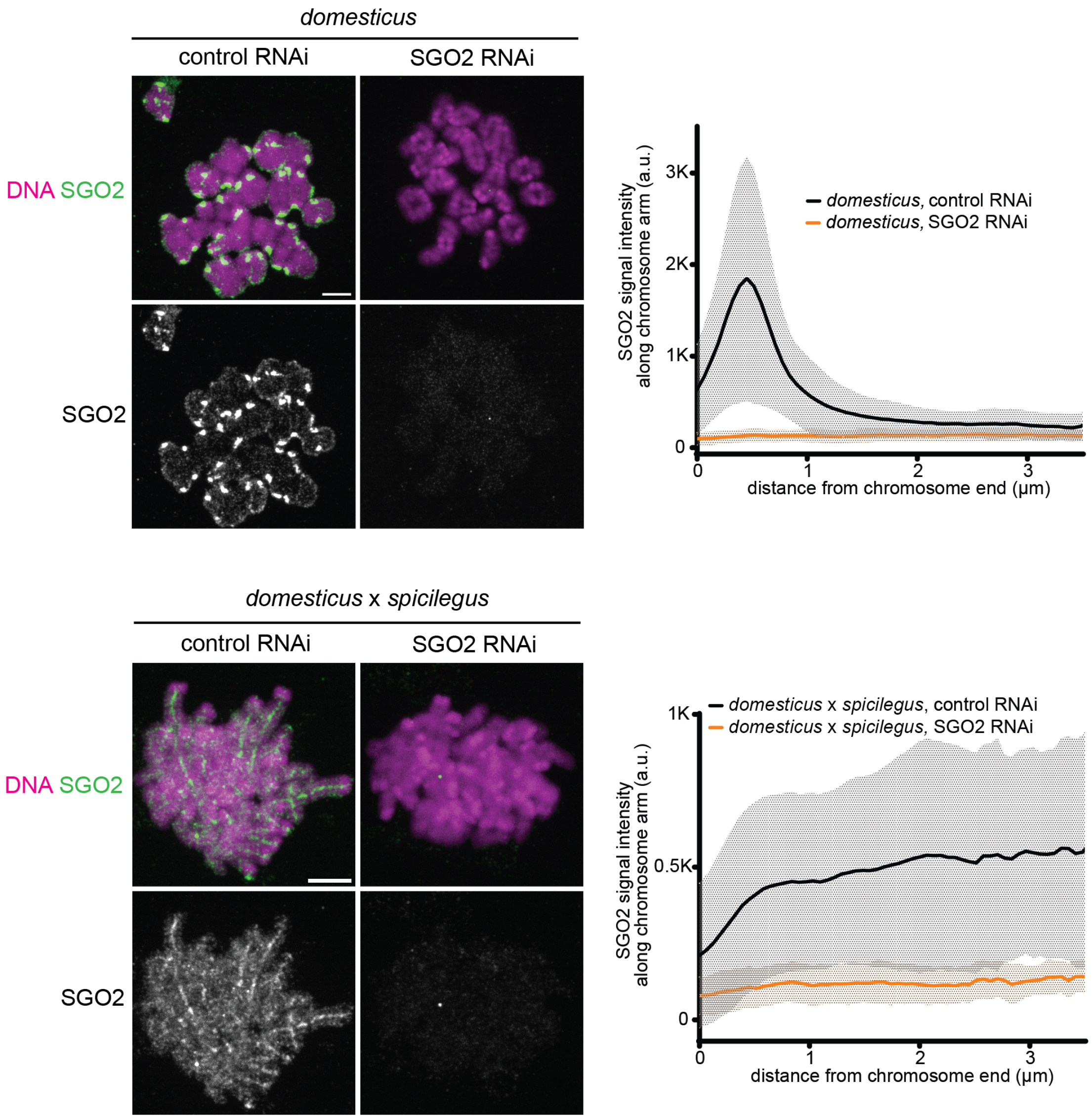

fig. S4. RNAi efficiently reduces SGO2 on the chromosomes. Chromosome spreads were performed from *domesticus* and hybrid oocytes electroporated with control or SGO2 siRNA were fixed at metaphase I and stained for SGO2. Line graphs are the quantification of SGO2 signal intensities along the chromosome arm starting from the centromere. Lines indicate the mean values of SGO2 intensity, and the shaded regions represents standard deviation (n = 138, 126, 105, and 94 chromosomes for *domesticus* + control RNAi, *domesticus* + SGO2 RNAi, hybrid + control RNAi, and hybrid + SGO2 RNAi, respectively); scale bars, 5 μm.

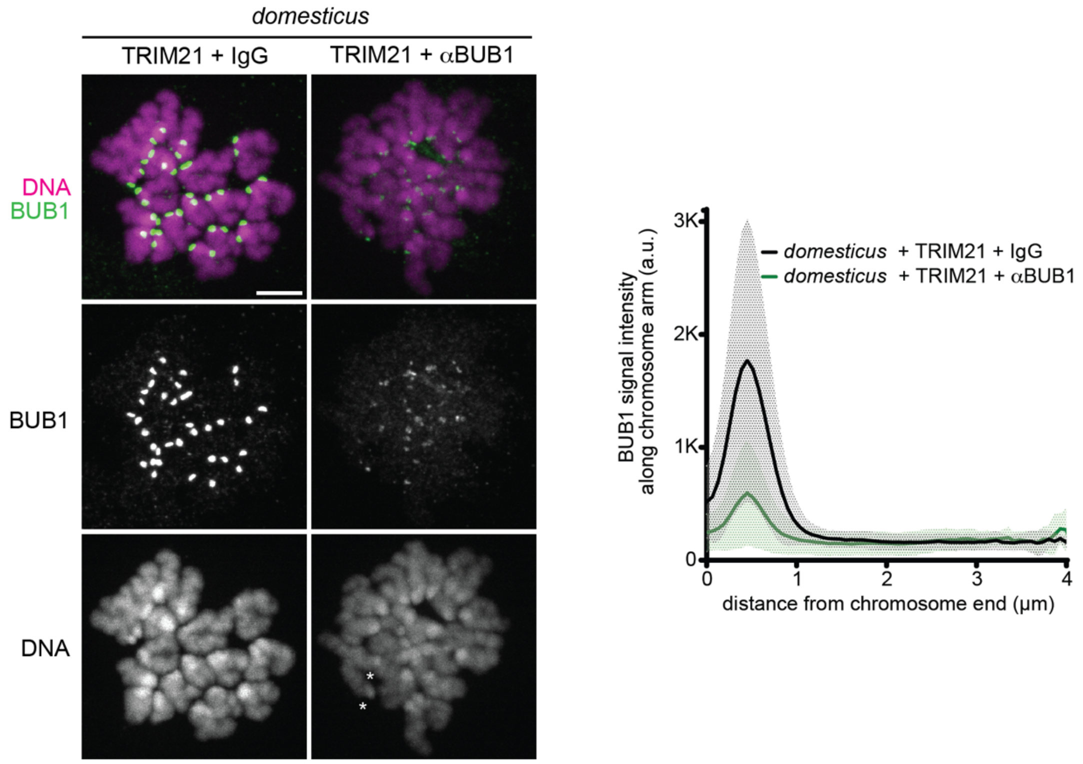
domesticus
TRIM21 + IgG
TRIM21 + αBUB1
DNA
BUB1
BUB1
DNA
*
*
3K
2K
1K
0
0
1
2
3
4
BUB1 signal intensity along chromosome arm (a.u.)
distance from chromosome end (µm)
domesticus + TRIM21 + IgG
domesticus + TRIM21 + αBUB1

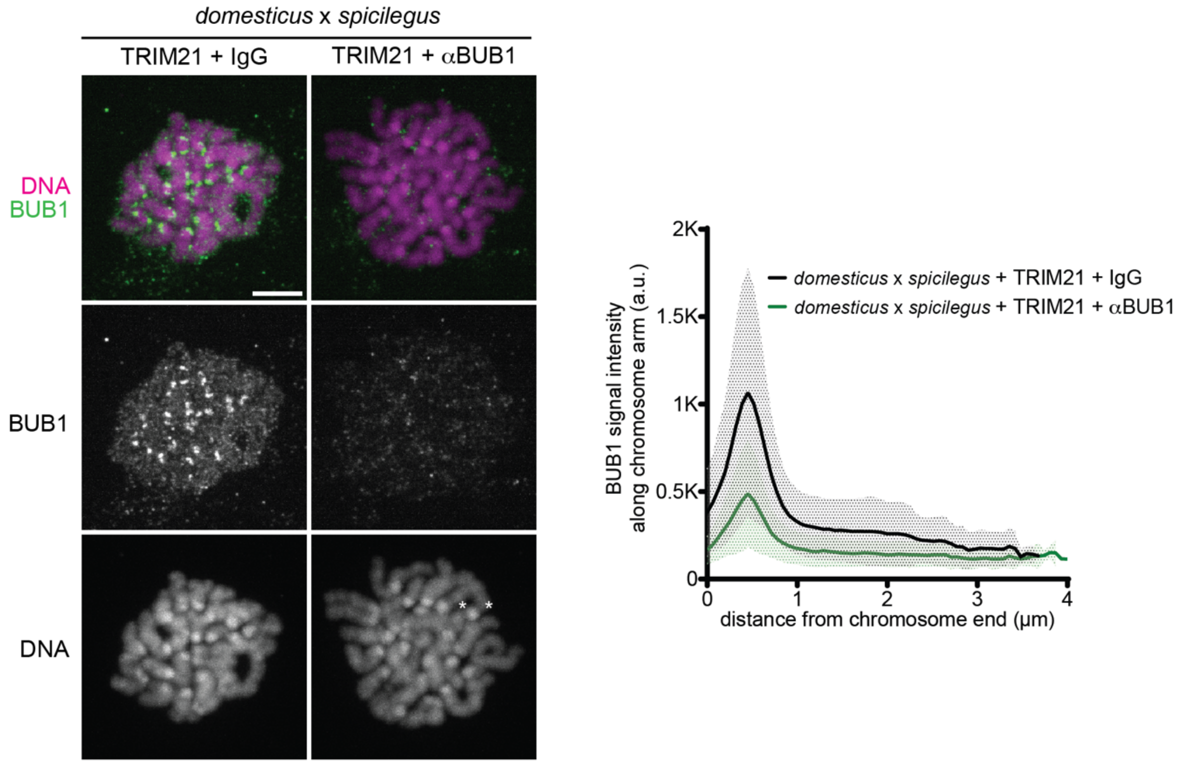
domesticus x spicilegus
TRIM21 + IgG
TRIM21 + αBUB1
DNA
BUB1
BUB1
DNA
* *
2K
1.5K
1K
0.5K
0
0
1
2
3
4
BUB1 signal intensity along chromosome arm (a.u.)
distance from chromosome end (µm)
domesticus x spicilegus + TRIM21 + IgG
domesticus x spicilegus + TRIM21 + αBUB1

fig. S5. BUB1 is efficiently reduced by Trim-Away. Chromosome spreads were performed from *domesticus* and hybrid oocytes expressing mCherry-TRIM21 with the control IgG or the anti-BUB1 antibody were fixed at metaphase II and stained for BUB1. Asterisks in the images denote sister chromatid precociously separated. Lines indicate the mean values of SGO2 intensity, and the shaded regions represents standard deviation (n = 126, 133, 116, and 118 chromosomes for *domesticus* +TRIM21 +IgG, *domesticus* +TRIM21 +anti-BUB1, hybrid +TRIM21 +IgG, and hybrid +TRIM21 +anti-BUB1, respectively); scale bars, 5 μm.

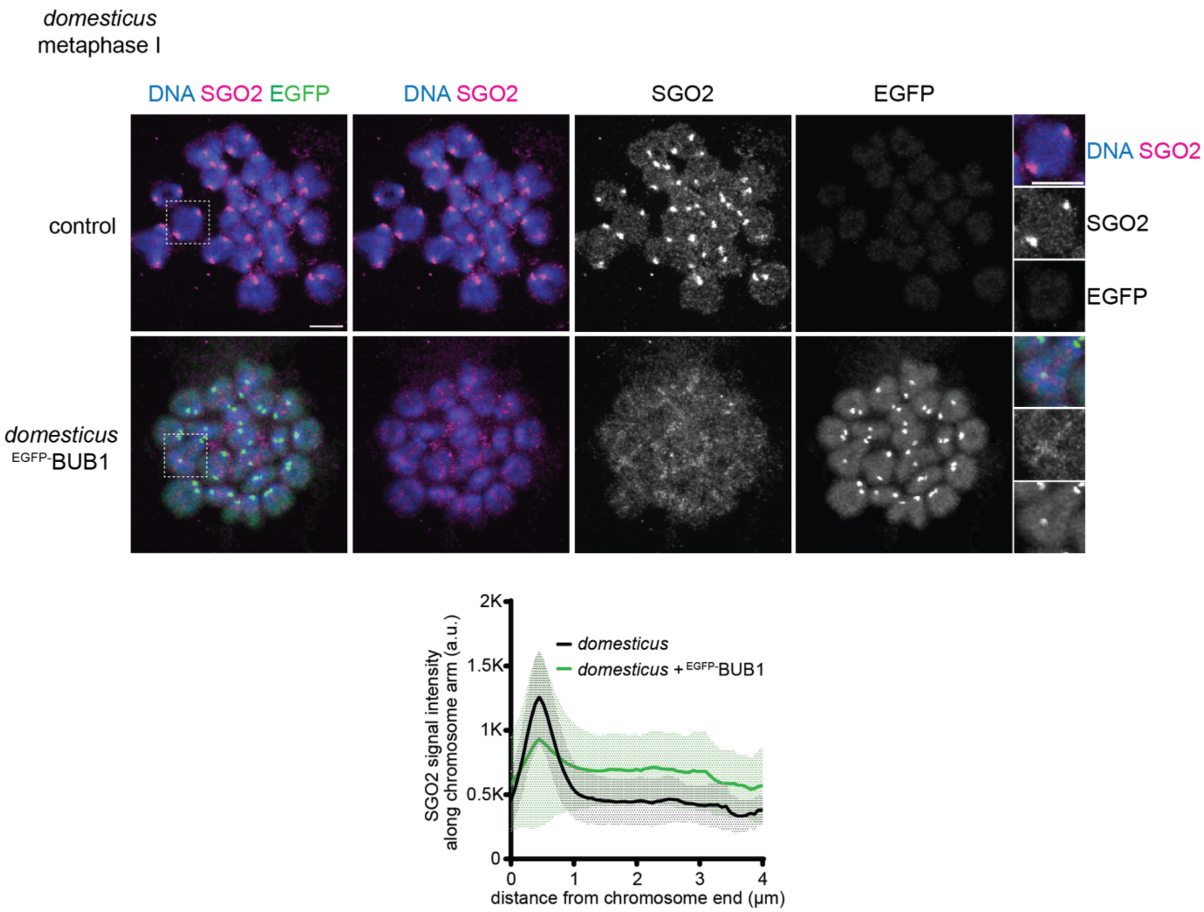

fig. S6. BUB1 overexpression increases SGO2 at chromosome arms. Chromosome spreads were performed from *domesticus* oocytes expressing EGFP-BUB1 were fixed at metaphase I and stained for GFP and SGO2. Lines indicate the mean values of SGO2 intensity, and the shaded regions represents standard deviation (n = 89 and 94, chromosomes for *domesticus* and *domesticus* +EGFP-BUB1, respectively); scale bars, 5 μm.

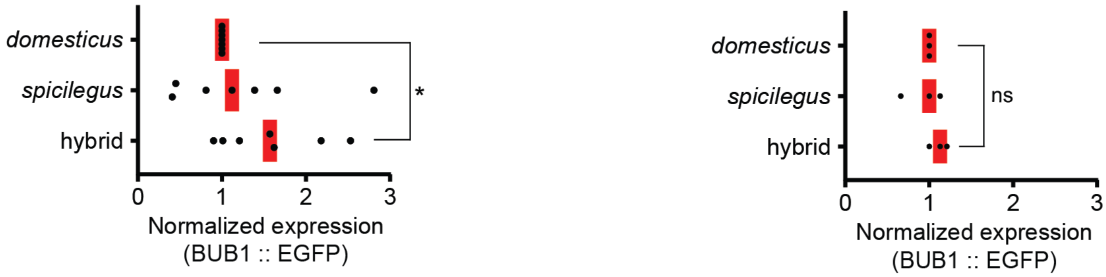

fig. S7. BUB1 transcripts are significantly higher in hybrid oocytes. Real-time qPCR results comparing relative abundance of BUB1 mRNA between *domesticus*, *spicilegus* and hybrid oocytes (left graph) and granulosa cells (right graph). EGFP was used as an exogenous control to normalize the total amount cDNA recovered by reverse transcription. Each dot in the graph represents one single experiment; red line, median; Mann-Whitney test was used for statistical analysis; $^{*}P < 0.05$.

Movie S1.

Example of a control *domesticus* oocyte undergoing anaphase I. DNA was visualized with SPY650-DNA. No obvious lagging chromosomes were observed.

Movie S2.

Example of a *domesticus* x *spicilegus* oocyte undergoing anaphase I. DNA was visualized with SPY650-DNA. Multiple chromosomes lagged during anaphase I.

Movie S3.

Example of a *domesticus* x *spicilegus* oocyte undergoing anaphase I. DNA was visualized with SPY650-DNA. Multiple chromosomes lagged during anaphase I followed by cytokinesis failure.
